# Durability as an index of endurance exercise performance: Methodological considerations

**DOI:** 10.1113/EP092120

**Published:** 2025-03-27

**Authors:** Ben Hunter, Ed Maunder, Andrew M. Jones, Gabriele Gallo, Daniel Muniz‐Pumares

**Affiliations:** ^1^ School of Human Sciences London Metropolitan University London UK; ^2^ Sports Performance Research Institute New Zealand Auckland University of Technology Auckland New Zealand; ^3^ Department of Public Health and Sport Sciences University of Exeter Medical School, St Luke's Campus Exeter UK; ^4^ Università degli Studi di Pavia Voghera Lombardy Italy; ^5^ Knowledgeiswatt SRLs Desio Monza e Brianza Italy; ^6^ School of Health, Medicine and Life Sciences University of Hertfordshire Hatfield UK

**Keywords:** endurance performance, exercise capacity, fatigue resistance

## Abstract

Endurance athletes routinely complete physiological assessments to predict performance, inform training programmes and monitor subsequent training adaptations. This profiling is typically performed with the athlete in a ‘fresh’ (i.e., rested) condition, but physiological profiling variables deteriorate during prolonged exercise. Durability has been defined as the resilience to the deterioration of physiological variables and performance during or following prolonged exercise. Herein, we review the current approaches to measure durability. The construction of the fatiguing protocol affects durability profiles, with greater relative intensity and duration resulting in more marked deterioration of baseline measures. The design of durability assessments should control for factors that could impact durability measurements, such as nutrition and environmental characteristics, to ensure that outcomes are repeatable and can be compared between athletes or over time in the same athlete. The selection of these parameters should be based on the proposed research question or applied context and take account of the training status of the athlete. Accordingly, this review highlights important considerations to ensure that protocols for profiling durability in research and applied practice are appropriate.

## Abstract (highlights)

- **What is the topic of this review?**
  The review discusses current approaches and essential considerations for assessing physiological durability.
- **What advances does it highlight?**
  The review summarizes various methodological approaches used to profile the resilience to deterioration of physiological variables during or after prolonged exercise, (i.e., durability). It highlights key considerations for durability profiling in research and applied settings, including exercise intensity, duration, nutritional and environmental factors, and the appropriateness of examining physiological decrements in the field or in the laboratory.

## 1 INTRODUCTION

Endurance sports are cornerstone events in the Summer Olympic Games, capturing the attention of millions of viewers, with athletes planning their training to achieve peak performance in their specialist event. Endurance performance is multifaceted and is affected by a range of physiological, biomechanical and psychological factors (di Prampero et al., 1986; Hoogkamer et al., 2017; Jones et al., 2021; Joyner et al., 2008, 2011; McCormick et al., 2015). Endurance is classically considered as the ability to sustain work rate over a period ranging from ∼10 min to several hours (Coyle et al., 1988). This review will focus primarily on prolonged endurance events, that is, those lasting >60 min.

Three physiological parameters have been used to explain much of the variation in prolonged endurance performance between athletes: maximum oxygen uptake (${\dot{V}}_{O_{2}\max}$); exercise economy; and the highest sustainable fraction of ${\dot{V}}_{O_{2}\max}$ for a given distance, which is, in turn, related to the transition between domains of exercise, or so‐called physiological ‘thresholds’ (di Prampero et al., 1986; Jones et al., 2021; Joyner, 1991; Joyner et al., 2008; Lazzer et al., 2012; Maunder et al., 2022; McLaughlin et al., 2010). Combined, these physiological parameters determine a ‘performance speed’ (di Prampero et al., 1986; Joyner, 1991), which accounts for ∼72% of the variation in marathon performance between athletes (di Prampero et al., 1986) and ∼88% of the variation in a 30 min time trial (TT) following 2 h of moderate cycling (Maunder et al., 2022).

Endurance athletes routinely measure the above physiological variables to predict performance, inform training programmes and monitor adaptations (Maunder et al., 2021). These variables are typically assessed in a well‐rested or ‘fresh’ condition, and athletes limit their training beforehand. However, physiological variables often deteriorate during prolonged exercise (Clark et al., 2018; Hamilton et al., 2024; Maunder et al., 2021; Stevenson et al., 2022). For example, relative to fresh conditions, ${\dot{V}}_{O_{2}\max}$ has been shown to decrease following a prolonged bout of running (Unhjem, 2024), a simulated half‐marathon (Dressendorfer, 1991), the first two segments of an Olympic triathlon (De Vito et al., 1995) and prolonged cycling (Bitel et al., 2024). Likewise, gross cycling efficiency (Passfield & Doust, 2000; Stevenson et al., 2022) and running economy (Brueckner et al., 1991; Sproule, 1998; Xu & Montgomery, 1995; Zanini et al., 2024) deteriorate following prolonged exercise. Intensity domain transitions, such as the first ventilatory threshold (VT_1_) (Gallo et al., 2024; Hamilton et al., 2024; Stevenson et al., 2022) or critical power (CP) (Clark et al., 2018, Clark, Vanhatalo, Thompson, Wylie, et al., 2019, Clark, Vanhatalo, Thompson, Joseph, et al., 2019), also decay by ∼10% following fatiguing exercise. Importantly, although a mean reduction of ∼10% at group mean level was evident across the studies conducted by Clark and colleagues, a range of ∼1%–31% was observed between individuals (Jones, 2023).

Similar heterogeneity has been reported when considering performance, including diminished TT power output (Hamilton et al., 2024; Ørtenblad et al., 2024; Passfield & Doust, 2000; Valenzuela et al., 2023) and peak power output (Clark, Vanhatalo, Thompson, Joseph, et al., 2019; Klaris et al., 2024; Spragg et al., 2024) following prolonged exercise. For example, following a ∼4 h workout, 20 and 6 min TT performance decreased by ∼2.9% (Valenzuela et al., 2023) and ∼10% (Ørtenblad et al., 2024), respectively, but in both studies the authors reported a large inter‐individual variability [range, −8.5% to +1.1% in the study by Valenzuela et al. (2023) and −31% to +1% in the study by Ørtenblad et al. (2024)]. Importantly, the ability to maintain power output following previous work is a key determinant of success in professional cycling (Van Erp et al., 2021).

In the same way that classical physiological parameters are associated with endurance exercise performance, the deterioration of external work rate over time is also underpinned by changes in the underlying physiology. The deterioration of peak oxygen uptake (${\dot{V}}_{O_{2}\mathrm{peak}}$) (Ørtenblad et al., 2024; Unhjem, 2024), gross efficiency (Passfield & Doust, 2000) and VT_1_ (Hamilton et al., 2024) are associated with a reduced capacity to produce high‐intensity efforts. The durability of VT_1_ has also been shown to affect the ability to sustain submaximal efforts (Gallo et al., 2024). Maintaining a metabolic steady state [i.e., exercise below CP or critical speed (CS)] for prolonged periods is crucial in endurance sports, such as marathon running (Jones & Vanhatalo, 2017). Indeed, faster marathoner runners complete races at a higher percentage of their CS (Smyth & Muniz‐Pumares, 2020; Smyth et al., 2022). Therefore, the ability to preserve, as far as possible, fresh‐state physiological parameters has important performance implications. Accordingly, durability, or resilience to prolonged exercise‐induced deteriorations in physiological profiling variables during prolonged exercise (Maunder et al., 2021), has been proposed as a fourth parameter of endurance exercise performance (Jones, 2023; Maunder et al., 2021). In the 2024 Paris Summer Olympics, the cycling road races were 273 and 158 km for the men and women, with winning times of 6:19:34 (h:min:s) and 3:59:23, respectively. In the marathon, the men's and women's gold medallists took 2:06:26 and 2:22:55 to complete the event, respectively. Given the duration of these events, durability has clear implications for performance in several Olympic events, in addition to long‐distance events, including long‐distance triathlon, ultramarathons and grand tour racing.

**FIGURE 1:**
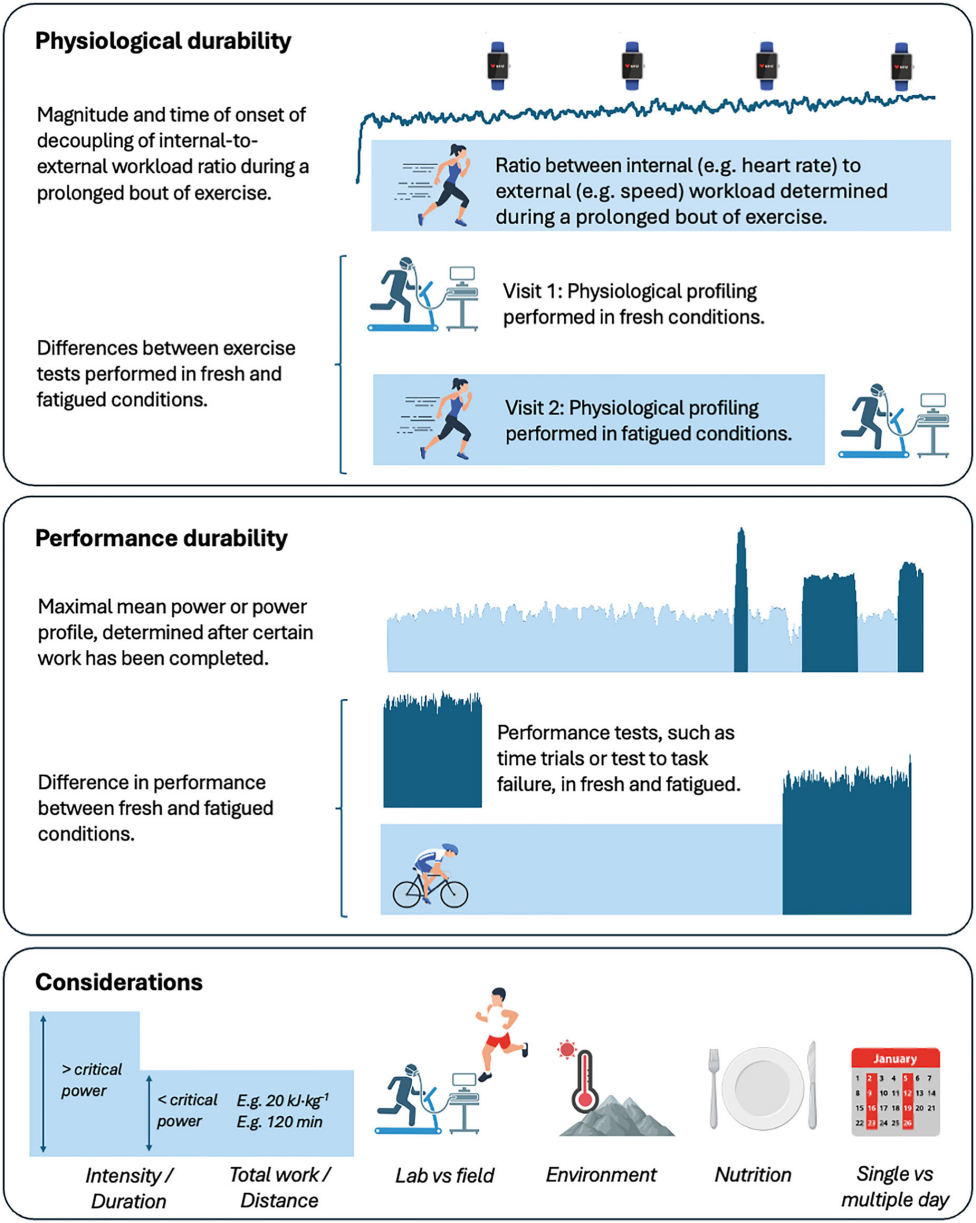
Summary of the different approaches to quantify durability (physiological and performance) and the considerations to be made when profiling durability.

**FIGURE 2:**
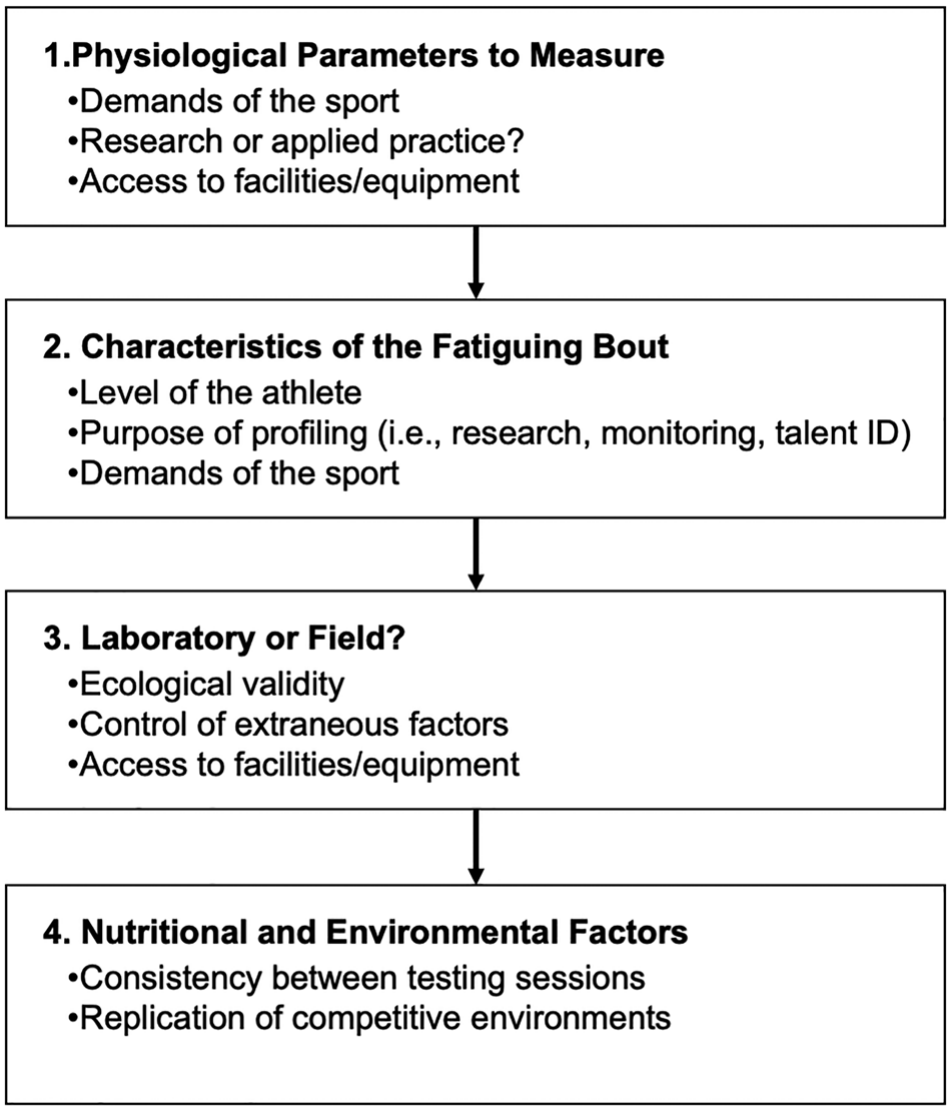
Flow chart outlining key considerations to be made when profiling durability.

Although protocols to assess ${\dot{V}}_{O_{2}\max}$ (Buchfuhrer et al., 1983; Poole & Jones, 2017), intensity domain transitions (Galán‐Rioja et al., 2020; Jamnick et al., 2020; Jones et al., 2019) and movement economy (Barnes & Kilding, 2015) have been discussed elsewhere, no consensus exists on durability assessment protocols. The overarching goal of this article is, therefore, to summarize current protocols to assess durability (Figure 1), with a predominant focus on physiological durability. Considerations relevant to the construction of the fatiguing protocol, and the conditions in which this protocol is performed, are also reviewed (Figure 2). We conclude by presenting key considerations to guide researchers and practitioners when designing protocols for the assessment of durability.

## 2 METHODOLOGICAL CONSIDERATIONS FOR ASSESSING DURABILITY

In this section, we discuss factors that could impact the outcome of durability assessments, including the structure of physiological measures (i.e., before and after, or during exercise), the intensity and duration of the fatiguing protocol, pre‐ and within‐exercise nutrition and environmental conditions (summarized in Figure 2). The factors that impact durability will be dependent on the setting (i.e., research or applied practice) and the sporting demands. Laboratory‐ or field‐based methods of assessing durability will also be discussed. Inadequate control of the considerations outlined below might result in spurious findings in a research context, or inadequate profiling of durability in an applied setting.

### 2.1 Structure of durability assessment

A multitude of different tests have been used to characterize physiological durability (Table 1). We identified two main methods by which this has been administered: (1) profiling before and after a prolonged bout of exercise; and (2) assessment of changes in physiological responses during prolonged exercise. These approaches can be combined by profiling responses before, during, and after a prolonged, fatiguing exercise bout.

**TABLE 1:** Summary of physiological profiling methods used in durability research and the number of testing days required to profile durability.

Type of test	Variables assessed	Sessions recommended for durability assessment
Submaximal incremental test	Moderate‐to‐heavy transition EE GE/RE Substrate oxidation rates	One
Maximal incremental test	Moderate‐to‐heavy transition Heavy‐to‐severe transition ${\dot{V}}_{O_{2}\max}$/${\dot{V}}_{O_{2}\mathrm{peak}}$ EE GE/RE Substrate oxidation rates Peak power/speed	Two
3MAOT	Heavy‐to‐severe transition ${\dot{V}}_{O_{2}\mathrm{peak}}$ *W*′ Peak power/speed Work performed/distance covered	Two
Time trial	${\dot{V}}_{O_{2}\mathrm{peak}}$ (if in severe domain) Peak power/speed Mean power/speed Work performed/distance covered	Two

Abbreviations: 3MAOT, 3 min all‐out test; EE, energy expenditure; GE, gross efficiency; RE, running economy; ${\dot{V}}_{O_{2}\max}$, maximum oxygen uptake; ${\dot{V}}_{O_{2}\mathrm{peak}}$, peak oxygen uptake; W', finite volume of work which can be expended above critical power.

#### 2.1.1 Before and after

Profiling before and after a prolonged bout of exercise can be done either on separate days or on the same day. A 2‐day protocol involves baseline profiling in the first session, followed by a repeat of the same tests immediately after prolonged exercise in a second session [e.g., a 3 min all‐out test; (Clark et al., 2018) or maximal incremental exercise test (Unhjem, 2024)]. Baseline measurements can also standardize the intensity of the prolonged exercise bout. A single‐day protocol involves physiological profiling immediately before and after a prolonged exercise bout (e.g., a submaximal incremental exercise test; Stevenson et al., 2022). This longer assessment may be preceded by an initial characterization trial. This can be used to improve the precision with which intensity domain transitions can be identified, particularly when using ventilatory and gas‐exchange data during single‐day assessments (Gallo et al., 2024; Stevenson et al., 2022). Changes in physiological parameters, expressed as delta values or percentage changes, can be used to evaluate physiological durability, with smaller relative or absolute differences from baseline indicating greater durability.

Measures of physiological durability during a single‐day assessment can be affected by the type of physiological tests performed (Table 1). For example, an incremental exercise test to determine ${\dot{V}}_{O_{2}\max}$ prior to and after prolonged exercise would incorporate time within the severe‐intensity domain, which would be expected to accelerate fatigue development (Black et al., 2017; Mateo‐March et al., 2024). This complicates distinguishing the effects of the incremental exercise test itself from those of the prolonged exercise, per se, on downward shifts in physiological parameters. The combination of transient (e.g., phosphocreatine (PCr) depletion; Harris et al., 1976) and sustained (e.g., glycogen depletion; Miura et al., 2000) effects of severe‐intensity exercise makes this approach problematic. Moreover, the restoration of physiological function following severe‐intensity exercise is influenced by the nature of recovery implemented (i.e., passive or active; e.g., Yoshida & Watari, 1996) and is likely to be impacted by inter‐individual variability in durability, which has been evidenced previously. Therefore, this approach does not enable confident quantification of changes to efficiency or economy during the prolonged trial. If researchers or practitioners wish to conduct assessments involving severe‐intensity exercise (e.g., maximal effort incremental tests, TTs or 3 min all‐out test), the baseline and postexercise assessments should be performed on separate days.

The durability of parameters quantified at lower intensities, such as the moderate‐to‐heavy intensity transition or efficiency, can be determined appropriately in one session. Following a characterization session, changes to VT_1_ have previously been demonstrated during one prolonged session (Gallo et al., 2024; Stevenson et al., 2022). Submaximal incremental tests induce less metabolic perturbation than higher‐intensity efforts (Black et al., 2017). Unlike an incremental exercise test to task failure, which can vary in length, by maintaining the same number of stages, this approach ensures a standardized volume of work done or distance covered. An initial characterization trial is usually required to prescribe the power output or speed of the prolonged trial and the submaximal incremental test stages. Alternatively, training data can inform the intensity of the fatiguing bout (Hunter et al., 2023; Karsten et al., 2015), reducing the time burden associated with assessing durability longitudinally. In an applied setting, this permits randomization of the testing sessions (if using a severe‐intensity effort) without the need for an additional characterization trial and would reduce the probability of a training effect occurring during durability profiling. However, in a research setting, characterization trials or baseline measures are essential for accurately identifying intensity domain transitions and prescribing the intensity of subsequent prolonged exercise. Further to these laboratory‐based approaches, field‐based methods provide more practical alternatives for assessing durability.

In field‐based settings, the power–duration or speed–duration relationship can be constructed from the best mean maximal power or speed thought to represent maximal efforts during training or TTs (Hunter et al., 2023; Karsten et al., 2015; Smyth & Muniz‐Pumares, 2020). These can be used to estimate CP and the finite volume of work that can be expended above CP (*W*′) prior to and after prolonged exercise (Spragg et al., 2024). The ‘gold‐standard’ protocol for estimating CP and *W*′ involves at least three exhaustive constant work‐rate bouts or maximal effort TTs, with additional trials conducted if the model fit is insufficient (Caen et al., 2024; Muniz‐Pumares et al., 2019). However, this protocol is cumbersome postexercise. Performing three or more trials within quick succession risks fatigue and insufficient *W*′ recovery (Muniz‐Pumares et al., 2019) or priming effects (Bailey et al., 2009), which will affect subsequent trials. Therefore, to construct the power–duration relationship when fresh and fatigued, six assessments would be necessary (i.e., three following prolonged exercise and three in a fresh condition), which is not practical in an applied setting.

Although using two trials to estimate CP and *W*′ with limited rest seems appealing, caution is warranted. The linear relationship using two data points will always result in a perfect fit (*R* ^2^ = 1.0), and no goodness‐of‐fit parameters (e.g., standard error of the estimate or coefficient of variation) can be derived. If the shorter‐duration trial exhibits a greater reduction relative to the longer‐duration trial following prolonged exercise, this could result in an artificial increase in the resultant CP and an underestimation of *W*′. Extending trial durations (i.e., TTs with a range of 7–20 min, instead of 2–15 min), might improve the accuracy of the 1/time model (Mattioni Maturana et al., 2018). Nonetheless, parameters from the power–duration relationship following prolonged exercise are prone to inaccuracies if methodologies diverge from recommended guidelines (Caen et al., 2024; Muniz‐Pumares et al., 2019).

In summary, although both single‐ and multi‐day protocols have the potential to provide valuable insights into durability, their suitability depends on the research question or applied context. Multi‐day protocols allow for more accurate assessments by reducing confounding factors, such as severe‐intensity fatigue accumulation, but they are resource intensive and less practical for longitudinal monitoring. Single‐day protocols, on the contrary, are more feasible and efficient, especially in applied settings, but require careful standardization of testing conditions to ensure reliable results.

#### 2.1.2 Measures during exercise

Further to assessing physiological function before and after prolonged exercise, physiological responses during prolonged exercise have been used to quantify durability. Durability has been quantified using decoupling of the internal‐to‐external work rate ratio (De Pauw et al., 2024; Maunder et al., 2021; Smyth et al., 2022). Initially, the ratio between internal work rate [e.g., rate of ventilation (*V̇*_E_) or heart rate (HR)] and external work rate (speed or power) is calculated early in a training session or competition (e.g., the 5–10 km segment of a marathon) and used as a baseline. An increase in this ratio relative to the baseline (i.e., decoupling) represents an increase in internal work rate for a given external work rate (speed or power), a decrease in external work rate for a given physiological strain, or a combination of both (De Pauw et al., 2024; Jones, 2023; Maunder et al., 2021; Smyth et al., 2022).

A recent study showed that recreational marathon runners with lower decoupling ratios completed marathons faster than those with higher ratios, and incorporating the magnitude and onset of decoupling reduced performance prediction error from ∼6.45% to ∼5.16% (Smyth et al., 2022). A similar approach has been used in runners when completing a ‘backyard ultra’ composed of 6.7 km laps, which started on the hour, every hour, until participants dropped out (De Pauw et al., 2024). Specifically, less proficient runners (i.e., those who completed <35 laps), exhibited significantly higher decoupling between HR and speed in comparison to more proficient runners (i.e., those who completed >35 laps). The magnitude of cardiovascular drift during 1 h of running at 70% of ${\dot{V}}_{O_{2}\max}$ has also been shown to be related to training status and to greater resilience of ${\dot{V}}_{O_{2}\max}$ and running economy (Unhjem, 2024). However, during a marathon, HR has been shown to dissociate from both ${\dot{V}}_{O_{2}\max}$ and the speed that elicits ${\dot{V}}_{O_{2}\max}$ (Billat et al., 2022). Therefore, HR might not provide an accurate reflection of metabolic cost during prolonged exercise, although it might still represent an increase in cardiac work. Limited research has demonstrated conflicting findings regarding the relationship between HR decoupling and deterioration of rested physiological parameters (Nuuttila et al., 2025; Unhjem, 2024). A greater magnitude of HR decoupling has been shown to be associated with decreases in ${\dot{V}}_{O_{2}\max}$ (Unhjem, 2024), but not with a loss of speed at the first lactate threshold (LT) (Nuuttila et al., 2025). Disparate mechanistic bases for the reductions in ${\dot{V}}_{O_{2}\max}$ and LT might explain the conflicting findings.

Heart rate variability (HRV), which is derived from the correlational properties of HR (i.e., detrended fluctuation analysis), might be a promising avenue for monitoring exercise intensity in the field (Gronwald & Hoos, 2020; Van Hooren et al., 2023). In fresh conditions, the fractal correlation properties of R–R interval data appear to be closely related to VT_1_ in running (Van Hooren et al., 2023) and cycling (Mateo‐March et al., 2023). Nuuttila et al. (2024) recently demonstrated an association between greater decreases in HRV during running and more marked deterioration of the speed at the first LT postexercise. However, the calculation of HRV is sensitive to artefacts, which might limit its practical application in the field. Non‐invasive wireless monitoring of muscle oxygenation has also been used to identify intensity domain transitions in rested conditions (Batterson et al., 2023). Encouragingly, the muscle oxygenation associated with the moderate‐to‐heavy transition was consistent between pre‐ and post‐measures following 150 min of moderate cycling (Hamilton et al., 2024). However, this was demonstrated only at the group level, and the within‐participant coefficient of variation was high (13%). Respiratory‐inductive plethysmography might also be an appropriate means of monitoring changes to *V̇*_E_ over time in accordance with changes to physiological thresholds (Stevenson et al., 2024). Indeed, the *V̇*_E_ associated with the moderate‐to‐heavy transition has been shown to remain unchanged following 90 min of running (Nuuttila et al., 2024) and 2 h of cycling (Stevenson et al., 2024). If *V̇*_E_ can be monitored accurately in real time, this could provide a means of assessing proximity to the moderate‐to‐heavy transition during prolonged exercise.

The increasing use of wearable technologies that are capable of monitoring physiological variables in real time and the ease of this approach makes decoupling a promising avenue to investigate durability. It remains unclear which marker or combination of markers of internal work rate [e.g., HR (Smyth et al., 2022), *V̇*_E_ (Stevenson et al., 2024) or muscle oxygenation (Hamilton et al., 2024)] should be used to assess physiological durability in the field, and what magnitude of decoupling should be exceeded to assume that physiological function has started to deteriorate. Therefore, future investigations are required to ascertain which decoupling measures reflect changes in physiological function during prolonged exercise.

### 2.2 Exercise intensity and duration

#### 2.2.1 Intensity

The intensity of prolonged exercise plays a crucial role in determining durability, with higher intensities leading to earlier and greater declines in physiological parameters, such as running economy (Howe et al., 2021; Unhjem, 2024; Xu & Montgomery, 1995; Zanini et al., 2024). The disparate magnitudes of metabolic disturbance between exercise intensity domains, particularly above CP, have been demonstrated previously (Black et al., 2017; Iannetta et al., 2022; Jones et al., 2007). Prescribing the intensity of prolonged exercise based on percentages of maximal values (e.g., ${\dot{V}}_{O_{2}\max}$), is questionable and should be discouraged (Iannetta et al., 2020; Lansley et al., 2011; Meyler et al., 2023). This is because, with such an approach, athletes can be operating in different intensity domains, each eliciting unique metabolic (Black et al., 2017; Iannetta et al., 2020; Lansley et al., 2011; Meyler et al., 2023) and neuromuscular (Brownstein et al., 2021) responses to exercise, which, in turn, can affect durability.

Recent research supports the notion that exercise intensity plays a crucial role in durability profiling, demonstrating that the amount of accumulated work above the LT is moderately related to the loss of TT power (Klaris et al., 2024). When considering differences between severe and moderate exercise intensities, bouts of either moderate intensity (70% of CP) or a repeated high‐intensity protocol (5 × 8 min at 105%–110% of CP) resulted in different levels of durability (Spragg et al., 2024). Indeed, most cyclists in the study were classified as ‘fatigable’, having exhibited reduced power outputs following both high‐ and low‐intensity protocols. However, 4 of the 13 cyclists demonstrated more pronounced reductions in power output following the high‐intensity protocol, with these reductions being more substantial during shorter duration power outputs. Likewise, Mateo‐March et al. (2024), found that even small amounts of work above CP (2.5 kJ kg^−1^) resulted in greater decreases of maximal mean power and CP when compared with work‐matched exercise performed below CP. MacDougall and colleagues (2024) provide further evidence for the role of greater metabolic disturbance in the reduction of performance. Performing high‐intensity exercise (10 × 2 min at 80% peak power) induces greater metabolic and contractile dysfunction when compared with low‐intensity constant work‐rate exercise. It has also been shown that heavy‐ but not moderate‐intensity exercise significantly reduces ${\dot{V}}_{O_{2}\mathrm{peak}}$ and peak power output during subsequent exercise (Brownstein et al., 2022).

Considering these findings, to ensure consistency in a research context, exercise should be standardized to intensity domain transitions. Previous studies used exercise intensity domains as a framework to regulate intensity during prolonged exercise (e.g., Clark et al., 2018; Clark, Vanhatalo, Thompson, Joseph, et al., 2019, Clark, Vanhatalo, Thompson, Wylie, et al., 2019; Gallo et al., 2024; Hamilton et al., 2024; Nuuttila et al., 2025; Spragg et al., 2024; Stevenson et al., 2022). Using a constant work rate permits repeated intermittent or continuous measurement of physiological data in a ‘steady state’ and can permit researchers to draw firmer conclusions about the aetiology of fatigue, and hence inferences about durability. However, constant work‐rate bouts do not adequately mimic the demands of competition, which tends to be stochastic in nature (Klaris et al., 2024; van Erp & Sanders, 2021).

In an applied context and in research where within‐participant variation is of interest, it might be more appropriate to exercise at an absolute power output or speed, for the same duration, to determine changes to durability over time (Matomäki et al., 2023). This method is standard practice when examining changes in running economy over time (e.g., Jones, 2006) and has been used to quantify changes in durability (Matomäki et al., 2023). Power or speed can be updated periodically in line with durability characteristics to ensure some deterioration in resting markers of performance or physiology. However, if a change to the speed or power corresponding to intensity domain transitions is expected, it is important to evaluate whether the speed or power of the fatiguing bout should be adjusted accordingly. This would ensure that improvements can be attributed to better durability rather than the work being completed at a lower relative intensity. Finally, for the purposes of talent identification, the approximate power output or speed expected within a competitive environment could be used for the prolonged bout. This would permit the identification of resilient or durable individuals who are able to maintain rested physiological parameters in the face of competitive demands.

#### 2.2.2 Duration and volume

Previous research has examined changes to physiological function over a range of time periods [e.g., 30 min (Fullerton et al., 2021) to 24 h (Gimenez et al., 2013)]. Clark, Vanhatalo, Thompson, Joseph, et al. (2019) demonstrated that 40 and 80 min of cycling in the heavy domain had no effect on CP, but after 120 min of cycling the CP decreased by ∼10% on average. A significant reduction of *W*′ from baseline occurred earlier, with a linear reduction that became significant after 80 min (Clark, Vanhatalo, Thompson, Joseph, et al., 2019). Likewise, power output at VT_1_ declined in a non‐linear fashion during prolonged cycling in the moderate domain (Gallo et al., 2024). Importantly, the onset of this decline differed substantially at the individual level and was related to the time to task failure. Therefore, when profiling durability, consideration should be given to the duration of the prolonged exercise bout.

Computing the total amount of work completed (or distance covered in running) accounts for both intensity and duration and thus appears to be an attractive candidate to standardize protocols. Several studies have taken this approach, particularly in cycling, where the power–duration profile has been determined in athletes after a certain amount of work has been accumulated (Mateo‐March et al., 2024, 2022; Muriel et al., 2022; Valenzuela et al., 2023). However, recent findings suggest that durability is more strongly influenced by the work performed in the heavy‐ and severe‐intensity domains rather than by the total accumulated work (Klaris et al., 2024; Leo et al., 2022; Spragg et al., 2024). When comparing durability between athletes with different baseline performance capacities, such as speed at LT or CP, comparisons based on specific distances or work completed can provide meaningful insights in a research setting. For example, if two runners of varying performance levels are prescribed a 90 min run at LT, the faster runner will cover a greater distance, potentially leading to greater deterioration in physiological parameters. Differences in durability profiles, such as running economy, have been observed between athletes when distance is the constant factor and intensity is matched (Zanini et al., 2024). This method might be less applicable when considering real‐world performance and might be more useful in a research setting. Notably, ‘highly successful’ athletes tend to show less pronounced declines in performance after fixed amounts of work in comparison to ‘less successful’ athletes (Van Erp et al., 2021). The timing of either performance bouts or physiological tests also warrant consideration. For sports that involve sporadic bouts of high‐intensity work (e.g., intermediate sprints) or very long exercise durations, it would be prudent to include multiple time points where durability can be profiled (e.g., Gallo et al., 2024; Klaris et al., 2024) This would enable practitioners to derive more specific profiles to inform pacing or other interventions.

In summary, the intensity and duration of the fatiguing bout are important factors that have been shown to influence the downward shift in performance and physiological parameters. The choice of these factors together should result in prolonged exercise that is demanding enough to observe changes from resting measures and will depend on the competitive level of the athlete (Van Erp et al., 2021). Failure to elicit a downward shift in physiological parameters from rest can result in an inability to draw meaningful conclusions about an intervention. For example, Sánchez‐Redondo et al. (2024) recently investigated the efficacy of caffeine ingestion on 8 min TT performance following 20 kJ kg^−1^ moderate‐intensity cycling. However, TT performance showed no significant differences between the caffeine and placebo conditions when compared with the fresh condition. As a result, the authors were not able to resolve the possible ergogenic effects of acute caffeine intake on TT performance following prolonged exercise. Pilot testing might be necessary to ensure that the fatiguing bout elicits a deterioration of the performance or physiological markers of interest. Given the propensity for some individuals to exhibit little change from baseline values after a fatiguing bout, pilot testing using a group can be useful to examine the range of responses. The duration of the bout should remain consistent between individuals and over time when testing longitudinally.

### 2.3 Nutritional availability

The energy demands of endurance events are satisfied primarily through oxidation of fats and carbohydrates. The relative contribution of fat and carbohydrate oxidation is, in turn, determined by the intensity and duration of exercise and can be influenced by nutritional strategies (Podlogar & Wallis, 2022; Romijn et al., 1993). It is plausible that nutritional status and, specifically, carbohydrate availability, plays a role in durability (Clark, Vanhatalo, Thompson, Joseph, et al., 2019; Dudley‐Rode et al., 2025; Ørtenblad et al., 2024; Spragg et al., 2024). For example, carbohydrate ingestion ameliorates the decline of CP (Clark, Vanhatalo, Thompson, Joseph, et al., 2019) and VT_1_ (Dudley‐Rode et al., 2025) during prolonged cycling. The rates of carbohydrate oxidation and fat oxidation have been implicated in the decline of CP and *W*′, but reductions in CP were not significantly correlated with changes in muscle glycogen concentration (Clark, Vanhatalo, Thompson, Joseph, et al., 2019). However, greater decreases in *W*′ were associated with more extensive glycogen depletion (Clark, Vanhatalo, Thompson, Joseph, et al., 2019). More recently, Ørtenblad et al. (2024) demonstrated weak associations between peak fat oxidation or fat oxidation over the first 3 h of exercise and durability in elite cyclists. However, higher fat oxidation rates in the fourth hour of exercise were associated with greater reduction of mean power output during a 6 min maximal TT following 4 h of cycling (Ørtenblad et al., 2024). The greater reliance on fat oxidation can be attenuated through strategies aimed at glycogen sparing (e.g., carbohydrate intake during exercise). Despite these findings, methodological differences regarding nutritional intake exist between studies. For example, in some studies athletes consumed carbohydrates during prolonged exercise (Bitel et al., 2024; Klaris et al., 2024; Ørtenblad et al., 2024; Spragg et al., 2024), whereas in other studies athletes were not allowed to consume carbohydrates (Clark et al., 2018; Stevenson et al., 2022; Unhjem, 2024; Zanini et al., 2024).

Carbohydrate ingestion during or before exercise reduces fat oxidation during exercise and reduces depletion of liver glycogen stores (Gonzalez et al., 2015; Jeukendrup et al., 2006; Podlogar & Wallis, 2022). Differences in substrate oxidation have previously been shown to be related to durability (Gallo et al., 2024; Ørtenblad et al., 2024; Spragg et al., 2023a). For example, the time to a 5% reduction in VT_1_ is associated with greater fat oxidation and lower carbohydrate oxidation rates during cycling (Gallo et al., 2024). Therefore, changes to diet or carbohydrate intake during exercise can hinder the ability to draw firm conclusions about the progress of athletes in applied practice or result in spurious findings in a research setting. It is recommended that carbohydrate ingestion, both before and during exercise, remains consistent between testing sessions. Despite the lack of empirical evidence, there is potential for other nutritional ergogenic aids to influence durability. For example, caffeine has been shown to improve prolonged cycling performance (Spriet et al., 1992) and 7 kJ kg^−1^ TT performance following prolonged cycling (Cox et al., 2002). Nitrate is a potential candidate for improving durability, because it attenuated the rise in oxygen consumption and the fall in muscle glycogen concentration during 2 h moderate‐intensity cycling, albeit without influencing performance in a subsequent 4 km TT (Tan et al., 2018). Nonetheless, given the potential for nutritional ergogenic aids to impact performance, it is recommended that the use of nutritional ergogenic aids is consistent between durability testing sessions. Finally, care should be taken to ensure that participants begin testing in a euhydrated state. It is recommended that athletes consume water ad libitum throughout durability assessments to mitigate negative effects of dehydration (Goulet & Hoffman, 2019).

### 2.4 Environment

Physiological testing is normally performed in temperature‐controlled laboratories, but athletic events often take place in more extreme environmental conditions. It is well established that altitude (Deb et al., 2018; Hamlin et al., 2015) and heat stress (Kuo et al., 2021; Wingo et al., 2020) have ergolytic effects on endurance performance. There is currently a lack of information regarding whether durability is also sensitive to environmental conditions and whether compromised durability plays a role in the decrease in performance observed during exercise in challenging environments. However, the negative impact of hypoxia appears to be greater over longer distances (Deb et al., 2018). When cycling and running in 35°C, greater cardiovascular strain and a larger reduction of ${\dot{V}}_{O_{2}\max}$ was observed after 45 min compared with 15 min (Wingo et al., 2020). However, similar repeated‐measures studies with a control condition in temperate environments are needed to evaluate the effects of thermal stress on durability. Some laboratories have incorporated physiological testing in temperate and extreme environmental conditions, which better represent the real racing conditions of athletes (e.g., Bell et al., 2017; Bourgois et al., 2023). Owing to the paucity of literature in this area, it is recommended that future research investigates the effects of environmental conditions on durability. In field studies, the control of environmental factors is difficult. For practitioners or researchers wishing to track durability over time, testing in similar environmental conditions is recommended to ensure that changes to durability are not influenced by differences in thermal stress. If researchers wish to compare means at the group level, care should be taken to ensure similar environmental conditions during testing in the field. If performance prediction is of importance, we advocate for testing to be conducted in conditions that correspond to those that are expected during the competition for which the training is undertaken.

### 2.5 Laboratory‐based versus field‐based measures

Field‐based measures allow data collection in ecologically valid environments, often requiring fewer resources, and can be used during training or competition. This approach permits ongoing monitoring of durability across a competitive season (Spragg et al., 2023b) and within races (De Pauw et al., 2024; Smyth et al., 2022) using wearable technology. For instance, retrospective analyses of power meter data have been performed to differentiate the performance category within a group of elite athletes (Van Erp et al., 2021). However, this method relies on maximal efforts being performed either naturally during training or deliberately for testing purposes. Conducting TTs both in a fresh condition and after accumulating work ensures the inclusion of maximal efforts in durability profiling, providing a more rigorous approach for estimating CP and *W*′ (Spragg et al., 2024). Another field‐based method, decoupling of the internal‐to‐external work rate ratio, has been proposed to quantify physiological drift (De Pauw et al., 2024; Maunder et al., 2021; Smyth et al., 2022). This could be achieved either during competition or during a controlled bout of exercise during training, to measure physiological drift (internal work rate) relative to speed or power (external work rate). TTs prior to and after accumulated work, or measurement of internal‐to‐external work rate, can both be executed during a controlled bout of exercise during training. Although such approaches facilitate longitudinal monitoring of durability, field‐based testing can be influenced by variables such as tactics, pacing strategies, motivation and environmental conditions. Additionally, during cycling, factors such as cadence (Barker et al., 2006), position (i.e., upright vs. TT position; Kordi et al., 2019) and terrain (i.e., flat vs. uphill; Nimmerichter et al., 2015) should be monitored or controlled during the trials to minimize variability in physiological responses.

In contrast, laboratory‐based testing provides a controlled environment ideal for examining the mechanistic underpinnings of durability. Researchers and practitioners can alter the metabolic perturbation precisely, by modifying duration (Clark, Vanhatalo, Thompson, Joseph, et al., 2019), intensity (Brownstein et al., 2022; Leo et al., 2022; Spragg et al., 2024) and nutritional intake (Clark, Vanhatalo, Thompson, Joseph, et al., 2019; Dudley‐Rode et al., 2025). Although this approach might lack ecological validity, if factors such as intensity and duration are not controlled, durability cannot be tracked accurately over time. Furthermore, alterations to hypoxic and environmental conditions are feasible to closely mimic competitive environments such as might be anticipated in, for example, the Summer Olympics. However, laboratory‐based tests can be problematic when conducting performance tests alongside durability measurements, particularly in running. TTs more closely replicate the demands of competition, whereas square‐wave time to task failure trials often lack specificity. Electromagnetically braked cycle ergometers or smart trainers permit the performance of TTs following a prolonged bout of exercise (Hamilton et al., 2024). Mixed reproducibility of TTs has been reported previously when performed on motorized treadmills (Doyle & Martinez, 1998; Russell et al., 2004; Schabort et al., 1998). To account for this, we recommend conducting familiarization trials if measures of performance are of interest in a laboratory setting. Furthermore, it might not be practical to test athletes after prolonged bouts of exercise to measure changes in physiological or performance in a laboratory setting owing to either time or resource constraints.

A potential avenue that has received little attention in the literature is to conduct the prolonged exercise bout in a quasi‐controlled environment outdoors before proceeding with laboratory‐based measurements (Bitel et al., 2024; Brueckner et al., 1991; Dressendorfer, 1991; Klaris et al., 2024). Cyclists recruited by Klaris et al. (2024) and Bitel et al. (2024) completed physiological profiling before and after 6 h and 90 min of outdoor cycling, respectively. Such an approach permits the rigour of laboratory‐based testing for the assessment of physiological phenomena alongside more ecologically valid fatiguing protocols. However, this method might not adequately replicate the profile of races or permit control of environmental factors. Moreover, and as noted by Bitel et al. (2024), the transition time from the fatiguing bout to subsequent testing might result in metabolic and energetic reconstitution. Shorter times to postexercise assessments have been associated with a much greater reduction in neuromuscular function (Brownstein et al., 2021). Therefore, the transition time from field to laboratory should be standardized between athletes and between visits to ensure consistency.

Predicting durability characteristics from traditional baseline measures offers a potentially time‐efficient alternative to laboratory‐based durability testing. Efforts in this area have yielded mixed results (Jones, 2023; Ørtenblad et al., 2024; Spragg et al., 2023a). For example, Spragg et al. (2023a) demonstrated excellent predictive capability of the change in CP, but not *W*′, following five 8 min efforts at 105%–110% CP using relative ${\dot{V}}_{O_{2}\max}$, gross efficiency, carbohydrate oxidation and fat oxidation in a multiple linear regression analysis. However, the change in CP following 2 h of heavy‐intensity cycling in the studies by Clark and colleagues (2018, 2019, 2019) was not significantly correlated with fresh measures of ${\dot{V}}_{O_{2}\max}$, gas exchange threshold, CP or *W*′ (Jones, 2023). Additionally, no significant correlations were reported between the change in mean 6 min power output after a 4 h intermittent cycling protocol and laboratory measures (i.e., LT, gross efficiency, fat oxidation and ${\dot{V}}_{O_{2}\mathrm{peak}}$; Ørtenblad et al., 2024). These discrepancies might stem from differences in fatiguing protocols and nutritional status, underlining the need for further research to clarify potential links between rested laboratory parameters and durability.

## 3 CONCLUSION

A large proportion of the observed inter‐individual variation in endurance exercise performance can be explained by ${\dot{V}}_{O_{2}\max}$, exercise economy and metabolic thresholds. However, these indices of endurance performance deteriorate with prolonged exercise and, therefore, performance capacity also decreases. Durability, which is defined as the ability to preserve physiological function during prolonged exercise, is an emerging component of endurance performance. Unlike traditional markers of endurance performance, the quantification of durability has not been standardized, and so far, durability has been evaluated through a range of physiological and performance approaches. Physiological approaches to evaluate durability include the assessment of physiological characteristics at rest and after prolonged exercise, or quantification of the decoupling of the internal‐to‐external work rate ratio during exercise.

In summary, factors such as exercise intensity and duration, nutritional status and environmental status have the potential to influence durability. The best practice for the profiling of durability will depend on the intended application and the context. We encourage practitioners and researchers to take account of the considerations highlighted in Figure 2 and discussed throughout this review, prior to profiling durability in athletes and study participants. We hope that greater rigour in the protocols used and more consistency in the approach taken by both researchers and applied practitioners in this emerging field will result in a greater collective understanding of the physiology underpinning durability during endurance exercise and the interventions that might enhance it.

## AUTHOR CONTRIBUTIONS

All authors have approved the final version of the manuscript and agree to be accountable for all aspects of the work in ensuring that questions related to the accuracy or integrity of any part of the work are appropriately investigated and resolved. All persons designated as authors qualify for authorship, and all those who qualify for authorship are listed.

## CONFLICT OF INTEREST

None declared.

## FUNDING INFORMATION

None.

## References

[eph13813-bib-0001] Bailey, S. J. , Vanhatalo, A. , Wilkerson, D. P. , Dimenna, F. J. , & Jones, A. M. (2009). Optimizing the “priming” effect: Influence of prior exercise intensity and recovery duration on O2 uptake kinetics and severe‐intensity exercise tolerance. Journal of Applied Physiology, 107(6), 1743–1756. 19797685 10.1152/japplphysiol.00810.2009

[eph13813-bib-0002] Barker, T. , Poole, D. C. , Noble, M. L. , & Barstow, T. J. (2006). Human critical power–oxygen uptake relationship at different pedalling frequencies. Experimental Physiology, 91(3), 621–632. 16527863 10.1113/expphysiol.2005.032789

[eph13813-bib-0003] Barnes, K. R. , & Kilding, A. E. (2015). Running economy: Measurement, norms, and determining factors. Sports Medicine Open, 1(1), 8. 27747844 10.1186/s40798-015-0007-y PMC4555089

[eph13813-bib-0004] Batterson, P. M. , Kirby, B. S. , Hasselmann, G. , & Feldmann, A. (2023). Muscle oxygen saturation rates coincide with lactate‐based exercise thresholds. European Journal of Applied Physiology, 123(10), 2249–2258. 37261552 10.1007/s00421-023-05238-9

[eph13813-bib-0005] Bell, P. G. , Furber, M. J. W. , van Someren, K. A. , Antón‐Solanas, A. , & Swart, J. (2017). The physiological profile of a multiple tour de france winning cyclist. Medicine and Science in Sports and Exercise, 49(1), 115–123. 27508883 10.1249/MSS.0000000000001068

[eph13813-bib-0006] Billat, V. , Palacin, F. , Poinsard, L. , Edwards, J. , & Maron, M. (2022). Heart rate does not reflect the %${\dot{V}}_{O_{2}\max}$ in recreational runners during the marathon. International Journal of Environmental Research and Public Health, 19(19), 12451. 36231750 10.3390/ijerph191912451 PMC9566186

[eph13813-bib-0007] Bitel, M. , Keir, D. A. , Grossman, K. , Barnes, M. , Murias, J. M. , & Belfry, G. R. (2024). The effects of a 90‐km outdoor cycling ride on performance outcomes derived from ramp‐incremental and 3‐minute all‐out tests. Journal of Strength and Conditioning Research, 38(3), 540–548. 38039445 10.1519/JSC.0000000000004650

[eph13813-bib-0008] Black, M. I. , Jones, A. M. , Blackwell, J. R. , Bailey, S. J. , Wylie, L. J. , McDonagh, S. T. J. , Thompson, C. , Kelly, J. , Sumners, P. , Mileva, K. N. , Bowtell, J. L. , & Vanhatalo, A. (2017). Muscle metabolic and neuromuscular determinants of fatigue during cycling in different exercise intensity domains. Journal of Applied Physiology, 122(3), 446–459. 28008101 10.1152/japplphysiol.00942.2016 PMC5429469

[eph13813-bib-0009] Bourgois, G. , Colosio, A. L. , Caen, K. , Bourgois, J. G. , Mucci, P. , & Boone, J. (2023). The effect of acute heat exposure on the determination of exercise thresholds from ramp and step incremental exercise. European Journal of Applied Physiology, 123(4), 847–856. 36507952 10.1007/s00421-022-05106-y

[eph13813-bib-0010] Brownstein, C. G. , Millet, G. Y. , & Thomas, K. (2021). Neuromuscular responses to fatiguing locomotor exercise. Acta Physiologica, 231(2). 10.1111/apha.13533 32627930

[eph13813-bib-0011] Brownstein, C. G. , Pastor, F. S. , Mira, J. , Murias, J. M. , & Millet, G. Y. (2022). Power output manipulation from below to above the gas exchange threshold results in exacerbated performance fatigability. Medicine and Science in Sports and Exercise, 54(11), 1947–1960. 36007155 10.1249/MSS.0000000000002976

[eph13813-bib-0012] Brueckner, J. C. , Atchou, G. , Capelli, C. , Duvallet, A. , Barrault, D. , Jousselin, E. , Rieu, M. , & di Prampero, P. E. (1991). The energy cost of running increases with the distance covered. European Journal of Applied Physiology and Occupational Physiology, 62(6), 385–389. 1893899 10.1007/BF00626607

[eph13813-bib-0013] Buchfuhrer, M. J. , Hansen, J. E. , Robinson, T. E. , Sue, D. Y. , Wasserman, K. , & Whipp, B. J. (1983). Optimizing the exercise protocol for cardiopulmonary assessment. Journal of Applied Physiology‐Respiratory, Environmental and Exercise Physiology, 55(5), 1558–1564. 6643191 10.1152/jappl.1983.55.5.1558

[eph13813-bib-0014] Caen, K. , Poole, D. C. , Vanhatalo, A. , & Jones, A. M. (2024). Critical power and maximal lactate steady state in cycling: “Watts” the difference? Sports Medicine, 54(10), 2497–2513. 39196486 10.1007/s40279-024-02075-4

[eph13813-bib-0015] Clark, I. E. , Vanhatalo, A. , Bailey, S. J. , Wylie, L. J. , Kirby, B. S. , Wilkins, B. W. , & Jones, A. M. (2018). Effects of two hours of heavy‐intensity exercise on the power‐duration relationship. Medicine and Science in Sports and Exercise, 50(8), 1658–1668. 29521722 10.1249/MSS.0000000000001601

[eph13813-bib-0016] Clark, I. E. , Vanhatalo, A. , Thompson, C. , Joseph, C. , Black, M. I. , Blackwell, J. R. , Wylie, L. J. , Tan, R. , Bailey, S. J. , Wilkins, B. W. , Kirby, B. S. , & Jones, A. M. (2019). Dynamics of the power‐duration relationship during prolonged endurance exercise and influence of carbohydrate ingestion. Journal of Applied Physiology, 127(3), 726–736. 31295069 10.1152/japplphysiol.00207.2019

[eph13813-bib-0017] Clark, I. E. , Vanhatalo, A. , Thompson, C. , Wylie, L. J. , Bailey, S. J. , Kirby, B. S. , Wilkins, B. W. , & Jones, A. M. (2019). Changes in the power‐duration relationship following prolonged exercise: Estimation using conventional and all‐out protocols and relationship with muscle glycogen. American Journal of Physiology‐Regulatory Integrative and Comparative Physiology, 317(1), R59–R67. 30995104 10.1152/ajpregu.00031.2019

[eph13813-bib-0018] Cox, G. R. , Desbrow, B. , Montgomery, P. G. , Anderson, M. E. , Bruce, C. R. , Macrides, T. A. , Martin, D. T. , Moquin, A. , Roberts, A. , Hawley, J. A. , & Burke, L. M. (2002). Effect of different protocols of caffeine intake on metabolism and endurance performance. Journal of Applied Physiology, 93(3), 990–999. 12183495 10.1152/japplphysiol.00249.2002

[eph13813-bib-0019] Coyle, E. F. , Coggan, A. R. , Hopper, M. K. , & Walters, T. J. (1988). Determinants of endurance in well‐trained cyclists. Journal of Applied Physiology, 64(6), 2622–2630. 3403447 10.1152/jappl.1988.64.6.2622

[eph13813-bib-0020] Deb, S. K. , Brown, D. R. , Gough, L. A. , Mclellan, C. P. , Swinton, P. A. , Andy Sparks, S. , & Mcnaughton, L. R. (2018). Quantifying the effects of acute hypoxic exposure on exercise performance and capacity: A systematic review and meta‐regression. European Journal of Sport Science, 18(2), 243–256. 29220311 10.1080/17461391.2017.1410233

[eph13813-bib-0021] De Pauw, K. , Ampe, T. , Arauz, Y. L. A. , Galloo, X. , Buyse, L. , Olieslagers, M. , Demuyser, T. , Corlùy, H. , Lamarti, S. , Provyn, S. , Jones, A. M. , Meeusen, R. , & Roelands, B. (2024). Backyard running: Pushing the boundaries of human performance. European Journal of Sport Science, 24(10), 1432–1441. 39276329 10.1002/ejsc.12190 PMC11451558

[eph13813-bib-0022] De Vito, G. , Bernardi, M. , Sproviero, E. , & Figura, F. (1995). Decrease of endurance performance during Olympic Triathlon. International Journal of Sports Medicine, 16(01), 24–28. 7713626 10.1055/s-2007-972958

[eph13813-bib-0023] di Prampero, P. E. , Atchou, G. , Brückner, J. C. , & Moia, C. (1986). The energetics of endurance running. European Journal of Applied Physiology and Occupational Physiology, 55(3), 259–266. 3732253 10.1007/BF02343797

[eph13813-bib-0024] Doyle, J. A. , & Martinez, A. L. (1998). Reliability of a protocol for testing endurance performance in runners and cyclists. Research Quarterly for Exercise and Sport, 69(3), 304–307. 9777668 10.1080/02701367.1998.10607698

[eph13813-bib-0025] Dressendorfer, R. H. (1991). Acute reduction in maximal oxygen uptake after long‐distance running. International Journal of Sports Medicine, 12(01), 30–33. 2030056 10.1055/s-2007-1024651

[eph13813-bib-0026] Dudley‐Rode, H. , Zinn, C. , Plews, D. J. , Charoensap, T. , & Maunder, E. (2025). Carbohydrate ingestion during prolonged exercise blunts the reduction in power output at the moderate‐to‐heavy intensity transition. European Journal of Applied Physiology, 125, 1349–1359. 39709586 10.1007/s00421-024-05687-w

[eph13813-bib-0027] Fullerton, M. M. , Passfield, L. , MacInnis, M. J. , Iannetta, D. , & Murias, J. M. (2021). Prior exercise impairs subsequent performance in an intensity‐ and duration‐dependent manner. Applied Physiology, Nutrition, and Metabolism, 46(8), 976–985. 10.1139/apnm-2020-0689 33641346

[eph13813-bib-0028] Galán‐Rioja, M. Á. , González‐Mohíno, F. , Poole, D. C. , & González‐Ravé, J. M. (2020). Relative proximity of critical power and metabolic/ventilatory thresholds: Systematic review and meta‐analysis. In Sports medicine. (Vol. 50, Issue (10), pp. 1771–1783) Springer. 32613479 10.1007/s40279-020-01314-8

[eph13813-bib-0029] Gallo, G. , Faelli, E. L. , Ruggeri, P. , Filipas, L. , Codella, R. , Plews, D. J. , & Maunder, E. (2024). Power output at the moderate‐to‐heavy intensity transition decreases in a non‐linear fashion during prolonged exercise. European Journal of Applied Physiology, 124(8), 2353–2364. 38483635 10.1007/s00421-024-05440-3 PMC11322563

[eph13813-bib-0030] Gimenez, P. , Kerhervé, H. , Messonnier, L. A. , Féasson, L. , & Millet, G. Y. (2013). Changes in the energy cost of running during a 24‐h treadmill exercise. Medicine and Science in Sports and Exercise, 45(9), 1807–1813. 23524515 10.1249/MSS.0b013e318292c0ec

[eph13813-bib-0031] Gonzalez, J. T. , Fuchs, C. J. , Smith, F. E. , Thelwall, P. E. , Taylor, R. , Stevenson, E. J. , Trenell, M. I. , Cermak, N. M. , & van Loon, L. J. C. (2015). Ingestion of glucose or sucrose prevents liver but not muscle glycogen depletion during prolonged endurance‐type exercise in trained cyclists. American Journal of Physiology‐Endocrinology and Metabolism, 309(12), E1032–E1039. 26487008 10.1152/ajpendo.00376.2015

[eph13813-bib-0032] Goulet, E. D. B. , & Hoffman, M. D. (2019). Impact of Ad libitum versus programmed drinking on endurance performance: A systematic review with meta‐analysis. Sports Medicine, 49(2), 221–232. 30659500 10.1007/s40279-018-01051-z

[eph13813-bib-0033] Gronwald, T. , & Hoos, O. (2020). Correlation properties of heart rate variability during endurance exercise: A systematic review. Annals of Noninvasive Electrocardiology, 25(1), e12697. 31498541 10.1111/anec.12697 PMC7358842

[eph13813-bib-0034] Hamilton, K. , Kilding, A. E. , Plews, D. J. , Mildenhall, M. J. , Waldron, M. , Charoensap, T. , Cox, T. H. , Brick, M. J. , Leigh, W. B. , & Maunder, E. (2024). Durability of the moderate‐to‐heavy‐intensity transition is related to the effects of prolonged exercise on severe‐intensity performance. European Journal of Applied Physiology, 124(8), 2427–2438. 38546844 10.1007/s00421-024-05459-6 PMC11322397

[eph13813-bib-0035] Hamlin, M. J. , Hopkins, W. G. , & Hollings, S. C. (2015). Effects of altitude on performance of elite track‐and‐field athletes. International Journal of Sports Physiology and Performance, 10(7), 881–887. 25710483 10.1123/ijspp.2014-0261

[eph13813-bib-0036] Harris, R. C. , Edwards, R. H. T. , Hultman, E. , Nordesjö, L. O. , Nylind, B. , & Sahlin, K. (1976). The time course of phosphorylcreatine resynthesis during recovery of the quadriceps muscle in man. Pflügers Archiv European Journal of Physiology, 367(2), 137–142. 1034909 10.1007/BF00585149

[eph13813-bib-0037] Hoogkamer, W. , Kram, R. , & Arellano, C. J. (2017). How biomechanical improvements in running economy could break the 2‐hour marathon barrier. Sports Medicine, 47(9), 1739–1750. 28255937 10.1007/s40279-017-0708-0

[eph13813-bib-0038] Howe, C. C. F. , Swann, N. , Spendiff, O. , Kosciuk, A. , Pummell, E. K. L. , & Moir, H. J. (2021). Performance determinants, running energetics and spatiotemporal gait parameters during a treadmill ultramarathon. European Journal of Applied Physiology, 121(6), 1759–1771. 33704547 10.1007/s00421-021-04643-2 PMC8144128

[eph13813-bib-0039] Hunter, B. , Ledger, A. , & Muniz‐Pumares, D. (2023). Remote determination of critical speed and critical power in recreational runners. International Journal of Sports Physiology and Performance, 18(12), 1449–1456. 37888148 10.1123/ijspp.2023-0276

[eph13813-bib-0040] Iannetta, D. , Inglis, E. C. , Mattu, A. T. , Fontana, F. Y. , Pogliaghi, S. , Keir, D. A. , & Murias, J. M. (2020). A critical evaluation of current methods for exercise prescription in women and men. Medicine and Science in Sports and Exercise, 52(2), 466–473. 31479001 10.1249/MSS.0000000000002147

[eph13813-bib-0041] Iannetta, D. , Zhang, J. , Murias, J. M. , & Aboodarda, S. J. (2022). Neuromuscular and perceptual mechanisms of fatigue accompanying task failure in response to moderate‐, heavy‐, severe‐, and extreme‐intensity cycling. Journal of Applied Physiology, 133(2), 323–334. 35771217 10.1152/japplphysiol.00764.2021

[eph13813-bib-0042] Jamnick, N. A. , Pettitt, R. W. , Granata, C. , Pyne, D. B. , & Bishop, D. J. (2020). An examination and critique of current methods to determine exercise intensity. Sports Medicine, 50(10), 1729–1756. 32729096 10.1007/s40279-020-01322-8

[eph13813-bib-0043] Jeukendrup, A. E. , Moseley, L. , Mainwaring, G. I. , Samuels, S. , Perry, S. , & Mann, C. H. (2006). Exogenous carbohydrate oxidation during ultraendurance exercise. Journal of Applied Physiology, 100(4), 1134–1141. 16322366 10.1152/japplphysiol.00981.2004

[eph13813-bib-0044] Jones, A. M. (2006). The physiology of the world record holder for the women's marathon. International Journal of Sports Science & Coaching, 1(2), 101–116.

[eph13813-bib-0045] Jones, A. M. (2023). The fourth dimension: Physiological resilience as an independent determinant of endurance exercise performance. The Journal of Physiology, 602(17), 4113–4128. 37606604 10.1113/JP284205

[eph13813-bib-0046] Jones, A. M. , Burnley, M. , Black, M. I. , Poole, D. C. , & Vanhatalo, A. (2019). The maximal metabolic steady state: Redefining the ‘gold standard’. Physiological Reports, 7(10), e14098. 31124324 10.14814/phy2.14098 PMC6533178

[eph13813-bib-0047] Jones, A. M. , Kirby, B. S. , Clark, I. E. , Rice, H. M. , Fulkerson, E. , Wylie, L. J. , Wilkerson, D. P. , Vanhatalo, A. , & Wilkins, B. W. (2021). Physiological demands of running at 2‐hour marathon race pace. Journal of Applied Physiology, 130(2), 369–379. 33151776 10.1152/japplphysiol.00647.2020

[eph13813-bib-0048] Jones, A. M. , & Vanhatalo, A. (2017). The ‘Critical Power’ concept: Applications to sports performance with a focus on intermittent high‐intensity exercise. Sports Medicine, 47(S1), 65–78. 28332113 10.1007/s40279-017-0688-0 PMC5371646

[eph13813-bib-0049] Jones, A. M. , Wilkerson, D. P. , DiMenna, F. , Fulford, J. , & Poole, D. C. (2007). Muscle metabolic responses to exercise above and below the “critical power” assessed using 31P‐MRS. American Journal of Pathology‐Regulatory, Integrative and Comparative Physiology, 294(2), R585–R593. 10.1152/ajpregu.00731.2007 18056980

[eph13813-bib-0050] Joyner, M. J. (1991). Modeling: Optimal marathon performance on the basis of physiological factors. Journal of Applied Physiology, 70(2), 683–687. 2022559 10.1152/jappl.1991.70.2.683

[eph13813-bib-0051] Joyner, M. J. , Coyle, E. F. , & Joyner, M. J. (2008). Endurance exercise performance: The physiology of champions. The Journal of Physiology, 586(1), 35–44. 17901124 10.1113/jphysiol.2007.143834 PMC2375555

[eph13813-bib-0052] Joyner, M. J. , Ruiz, J. R. , & Lucia, A. (2011). The two‐hour marathon: Who and when? Journal of Applied Physiology, 110(1), 275–277. 20689089 10.1152/japplphysiol.00563.2010

[eph13813-bib-0053] Karsten, B. , Jobson, S. A. , Hopker, J. , Stevens, L. , & Beedie, C. (2015). Validity and reliability of critical power field testing. European Journal of Applied Physiology, 115(1), 197–204. 25260244 10.1007/s00421-014-3001-z

[eph13813-bib-0054] Klaris, M. B. , Cubel, C. , Bruun, T. R. , Stampe, D. , Rørvik, S. , Fischer, M. , Bonne, T. , Christensen, P. M. , Piil, J. F. , & Nybo, L. (2024). Performance and fatigue patterns in elite cyclists during 6 h of simulated road racing. Scandinavian Journal of Medicine and Science in Sports, 34(7), e14699. 39011951 10.1111/sms.14699

[eph13813-bib-0055] Kordi, M. , Fullerton, C. , Passfield, L. , & Parker Simpson, L. (2019). Influence of upright versus time trial cycling position on determination of critical power and W’ in trained cyclists. European Journal of Sport Science, 19(2), 192–198. 30009673 10.1080/17461391.2018.1495768

[eph13813-bib-0056] Kuo, Y.‐H. , Cheng, C.‐F. , Kuo, Y.‐C. , Marín, D. M. , Coll, J. S. , & Sánchez, I. B. (2021). Determining validity of critical power estimated using a three‐minute all‐out test in hot environments. International Journal of Environmental Research and Public Health, 18(17), 9193. 34501781 10.3390/ijerph18179193 PMC8431074

[eph13813-bib-0057] Lansley, K. E. , Dimenna, F. J. , Bailey, S. J. , & Jones, A. M. (2011). A “new” method to normalise exercise intensity. International Journal of Sports Medicine, 32(7), 535–541. 21563028 10.1055/s-0031-1273754

[eph13813-bib-0058] Lazzer, S. , Salvadego, D. , Rejc, E. , Buglione, A. , Antonutto, G. , & di Prampero, P. E. (2012). The energetics of ultra‐endurance running. European Journal of Applied Physiology, 112(5), 1709–1715. 21881950 10.1007/s00421-011-2120-z

[eph13813-bib-0059] Leo, P. , Giorgi, A. , Spragg, J. , Gonzalez, B. M. , & Mujika, I. (2022). Impact of prior accumulated work and intensity on power output in elite/international level road cyclists—a pilot study. German Journal of Exercise and Sport Research, 52(4), 673–677.

[eph13813-bib-0059a] MacDougall, K. B. , Zhang, J. , Grunau, M. , Anklovitch, E. , MacIntosh, B. R. , MacInnis, M. J. , & Aboodarda, S. J. (2024). Acute performance fatigability following continuous versus intermittent cycling protocols is not proportional to total work done. Applied Physiology, Nutrition, and Metabolism, 49(8), 1055–1067. 10.1139/apnm-2023-0503 38631044

[eph13813-bib-0060] Mateo‐March, M. , Leo, P. , Muriel, X. , Javaloyes, A. , Mujika, I. , Barranco‐Gil, D. , Pallarés, J. G. , Lucia, A. , & Valenzuela, P. L. (2024). Is all work the same? Performance after accumulated work of differing intensities in male professional cyclists. Journal of Science and Medicine in Sport, 27(6), 430–434. 38604818 10.1016/j.jsams.2024.03.005

[eph13813-bib-0061] Mateo‐March, M. , Moya‐Ramón, M. , Javaloyes, A. , Sánchez‐Muñoz, C. , & Clemente‐Suárez, V. J. (2023). Validity of detrended fluctuation analysis of heart rate variability to determine intensity thresholds in elite cyclists. European Journal of Sport Science, 23(4), 580–587. 35238695 10.1080/17461391.2022.2047228

[eph13813-bib-0062] Mateo‐March, M. , Valenzuela, P. L. , Muriel, X. , Gandia‐Soriano, A. , Zabala, M. , Lucia, A. , Pallares, J. G. , & Barranco‐Gil, D. (2022). The record power profile of male professional cyclists: Fatigue matters. International Journal of Sports Physiology and Performance, 17(6), 926–931. 35240578 10.1123/ijspp.2021-0403

[eph13813-bib-0063] Matomäki, P. , Heinonen, O. J. , Nummela, A. , Laukkanen, J. , Auvinen, E. P. , Pirkola, L. , & Kyröläinen, H. (2023). Durability is improved by both low and high intensity endurance training. Frontiers in Physiology, 14, 1128111. 36875044 10.3389/fphys.2023.1128111 PMC9977827

[eph13813-bib-0064] Mattioni Maturana, F. , Fontana, F. Y. , Pogliaghi, S. , Passfield, L. , & Murias, J. M. (2018). Critical power: How different protocols and models affect its determination. Journal of Science and Medicine in Sport, 21(7), 742–747. 29203319 10.1016/j.jsams.2017.11.015

[eph13813-bib-0065] Maunder, E. , Plews, D. J. , Wallis, G. A. , Brick, M. J. , Leigh, W. B. , Chang, W. L. , Stewart, T. , Watkins, C. M. , & Kilding, A. E. (2022). Peak fat oxidation is positively associated with vastus lateralis CD36 content, fed‐state exercise fat oxidation, and endurance performance in trained males. European Journal of Applied Physiology, 122(1), 93–102. 34562114 10.1007/s00421-021-04820-3 PMC8475903

[eph13813-bib-0066] Maunder, E. , Seiler, S. , Mildenhall, M. J. , Kilding, A. E. , & Plews, D. J. (2021). The importance of ‘Durability’ in the physiological profiling of endurance athletes. Sports Medicine, 51(8), 1619–1628. 33886100 10.1007/s40279-021-01459-0

[eph13813-bib-0067] Mccormick, A. , Meijen, C. , & Marcora, S. (2015). Psychological determinants of whole‐body endurance performance. Sports Medicine, 45(7), 997–1015. 25771784 10.1007/s40279-015-0319-6 PMC4473096

[eph13813-bib-0068] McLaughlin, J. E. , Howley, E. T. , Bassett, D. R. , Thompson, D. L. , & Fitzhugh, E. C. (2010). Test of the classic model for predicting endurance running performance. Medicine and Science in Sports and Exercise, 42(5), 991–997. 19997010 10.1249/MSS.0b013e3181c0669d

[eph13813-bib-0069] Meyler, S. , Bottoms, L. , Wellsted, D. , & Muniz‐Pumares, D. (2023). Variability in exercise tolerance and physiological responses to exercise prescribed relative to physiological thresholds and to maximum oxygen uptake. Experimental Physiology, 108(4), 581–594. 36710454 10.1113/EP090878 PMC10103872

[eph13813-bib-0070] Miura, A. , Sato, H. , Sato, H. , Whipp, B. J. W. , & Fukuba, Y. (2000). The effect of glycogen depletion on the curvature constant parameter of the power‐duration curve for cycle ergometry. Ergonomics, 43(1), 133–141. 10661696 10.1080/001401300184693

[eph13813-bib-0071] Muniz‐Pumares, D. , Karsten, B. , Triska, C. , & Glaister, M. (2019). Methodological approaches and related challenges associated with the determination of critical power and curvature constant. Journal of Strength and Conditioning Research, 33(2), 584–596. 30531413 10.1519/JSC.0000000000002977

[eph13813-bib-0072] Muriel, X. , Mateo‐March, M. , Valenzuela, P. L. , Zabala, M. , Lucia, A. , Pallares, J. G. , & Barranco‐Gil, D. (2022). Durability and repeatability of professional cyclists during a Grand Tour. European Journal of Sport Science, 22(12), 1797–1804. 34586952 10.1080/17461391.2021.1987528

[eph13813-bib-0073] Nimmerichter, A. , Prinz, B. , Haselsberger, K. , Novak, N. , Simon, D. , & Hopker, J. G. (2015). Gross efficiency during flat and uphill cycling in field conditions. International Journal of Sports Physiology and Performance, 10(7), 830–834. 25611890 10.1123/ijspp.2014-0373

[eph13813-bib-0074] Nuuttila, O‐P. , Laatikainen‐Raussi, V. , Vohlakari, K. , Laatikainen‐Raussi, I. , & Ihalainen, J. K. (2025). Durability in recreational runners: Effects of 90‐min low‐intensity exercise on the running speed at the lactate threshold. European Journal of Applied Physiology, 125, 697–705. 39384626 10.1007/s00421-024-05631-y PMC11889008

[eph13813-bib-0075] Ørtenblad, N. , Zachariassen, M. , Nielsen, J. , & Gejl, K. D. (2024). Substrate utilization and durability during prolonged intermittent exercise in elite road cyclists. European Journal of Applied Physiology, 124(7), 2193–2205. 38441690 10.1007/s00421-024-05437-y PMC11199313

[eph13813-bib-0076] Passfield, L. , & Doust, J. H. (2000). Changes in cycling efficiency and performance after endurance exercise. Medicine and Science in Sports and Exercise, 32(11), 1935–1941. 11079525 10.1097/00005768-200011000-00018

[eph13813-bib-0077] Podlogar, T. , & Wallis, G. A. (2022). New horizons in carbohydrate research and application for endurance athletes. Sports Medicine, 52(S1), 5–23. 36173597 10.1007/s40279-022-01757-1 PMC9734239

[eph13813-bib-0078] Poole, D. C. , & Jones, A. M. (2017). Measurement of the maximum oxygen uptake ${\dot{V}}_{O_{2}\max}$: ${\dot{V}}_{O_{2}\mathrm{peak}}$ is no longer acceptable. Journal of Applied Physiology, 122(4), 997–1002. 28153947 10.1152/japplphysiol.01063.2016

[eph13813-bib-0079] Romijn, J. A. , Coyle, E. F. , Sidossis, L. S. , Gastaldelli, A. , Horowitz, J. F. , Endert, E. , & Wolfe, R. R. (1993). Regulation of endogenous fat and carbohydrate metabolism in relation to exercise intensity and duration. The American Journal of Physiology, 265(3 Pt 1), E380–E391. 8214047 10.1152/ajpendo.1993.265.3.E380

[eph13813-bib-0080] Russell, R. D. , Redmann, S. M. , Ravussin, E. , Hunter, G. R. , & Larson‐Meyer, D. E. (2004). Reproducibility of endurance performance on a treadmill using a preloaded time trial. Medicine and Science in Sports and Exercise, 36(4), 717–724. 15064600 10.1249/01.mss.0000121954.95892.c8

[eph13813-bib-0081] Sánchez‐Redondo, I. R. , Alejo, L. B. , Revuelta, C. , de Pablos, R. , Ibañez, M. , Pérez‐López, A. , Lucia, A. , Barranco‐Gil, D. , & Valenzuela, P. L. (2024). Intrasession caffeine intake and cycling performance after accumulated work: A field‐based study. International Journal of Sport Nutrition and Exercise Metabolism, 35(1), 61–66. 39326860 10.1123/ijsnem.2024-0109

[eph13813-bib-0082] Schabort, E. J. , Hopkins, W. G. , & Hawley, J. A. (1998). Reproducibility of self‐paced treadmill performance of trained endurance runners. International Journal of Sports Medicine, 19(01), 48–51. 9506800 10.1055/s-2007-971879

[eph13813-bib-0083] Smyth, B. , Maunder, E. , Meyler, S. , Hunter, B. , & Muniz‐Pumares, D. (2022). Decoupling of internal and external workload during a marathon: An analysis of durability in 82,303 recreational runners. Sports Medicine, 52(9), 2283–2295. 35511416 10.1007/s40279-022-01680-5 PMC9388405

[eph13813-bib-0084] Smyth, B. , & Muniz‐Pumares, D. (2020). Calculation of critical speed from raw training data in recreational marathon runners. Medicine and Science in Sports and Exercise, 52(12), 2637–2645. 32472926 10.1249/MSS.0000000000002412 PMC7664951

[eph13813-bib-0085] Spragg, J. , Leo, P. , Giorgi, A. , Gonzalez, B. M. , & Swart, J. (2024). The intensity rather than the quantity of prior work determines the subsequent downward shift in the power duration relationship in professional cyclists. European Journal of Sport Science, 24(4), 449–457.

[eph13813-bib-0086] Spragg, J. , Leo, P. , & Swart, J. (2023a). The relationship between physiological characteristics and durability in male professional cyclists. Medicine and Science in Sports and Exercise, 55(1), 133–140. 35977108 10.1249/MSS.0000000000003024

[eph13813-bib-0087] Spragg, J. , Leo, P. , & Swart, J. (2023b). The relationship between training characteristics and durability in professional cyclists across a competitive season. European Journal of Sport Science, 23(4), 489–498. 35239466 10.1080/17461391.2022.2049886

[eph13813-bib-0088] Spriet, L. L. , MacLean, D. A. , Dyck, D. J. , Hultman, E. , Cederblad, G. , & Graham, T. E. (1992). Caffeine ingestion and muscle metabolism during prolonged exercise in humans. The American Journal of Physiology, 262(6 Pt 1), E891–E898. 1616022 10.1152/ajpendo.1992.262.6.E891

[eph13813-bib-0089] Sproule, J. (1998). Running economy deteriorates following 60 min of exercise at 80% ${\dot{V}}_{O_{2}\max}$ . European Journal of Applied Physiology and Occupational Physiology, 77(4), 366–371. 9562366 10.1007/s004210050346

[eph13813-bib-0090] Stevenson, J. D. , Kilding, A. E. , Plews, D. J. , & Maunder, E. (2022). Prolonged cycling reduces power output at the moderate‐to‐heavy intensity transition. European Journal of Applied Physiology, 122(12), 2673–2682. 36127418 10.1007/s00421-022-05036-9 PMC9488873

[eph13813-bib-0091] Stevenson, J. D. , Kilding, A. E. , Plews, D. J. , & Maunder, E. (2024). Prolonged exercise shifts ventilatory parameters at the moderate‐to‐heavy intensity transition. European Journal of Applied Physiology, 124(1), 309–315. 37495864 10.1007/s00421-023-05285-2 PMC10786968

[eph13813-bib-0092] Tan, R. , Wylie, L. J. , Thompson, C. , Blackwell, J. R. , Bailey, S. J. , Vanhatalo, A. , & Jones, A. M. (2018). Beetroot juice ingestion during prolonged moderate‐intensity exercise attenuates progressive rise in O2 uptake. Journal of Applied Physiology, 124(5), 1254–1263. 29357494 10.1152/japplphysiol.01006.2017

[eph13813-bib-0093] Unhjem, R. J. (2024). Changes in running economy and attainable maximal oxygen consumption in response to prolonged running: The impact of training status. Scandinavian Journal of Medicine and Science in Sports, 34(5), e14637. 38671555 10.1111/sms.14637

[eph13813-bib-0094] Valenzuela, P. L. , Alejo, L. B. , Ozcoidi, L. M. , Lucia, A. , Santalla, A. , & Barranco‐Gil, D. (2023). Durability in professional cyclists: A field study. International Journal of Sports Physiology and Performance, 18(1), 99–103. 36521188 10.1123/ijspp.2022-0202

[eph13813-bib-0095] van Erp, T. , & Sanders, D. (2021). Demands of professional cycling races: Influence of race category and result. European Journal of Sport Science, 21(5), 666–677. 32584197 10.1080/17461391.2020.1788651

[eph13813-bib-0096] Van Erp, T. , Sanders, D. , & Lamberts, R. P. (2021). Maintaining power output with accumulating levels of work done is a key determinant for success in professional cycling. Medicine and Science in Sports and Exercise, 53(9), 1903–1910. 33731651 10.1249/MSS.0000000000002656

[eph13813-bib-0097] Van Hooren, B. , Mennen, B. , Gronwald, T. , Bongers, B. C. , & Rogers, B. (2023). Correlation properties of heart rate variability to assess the first ventilatory threshold and fatigue in runners. Journal of Sports Sciences, 43(2), 125–134. 37916488 10.1080/02640414.2023.2277034

[eph13813-bib-0098] Wingo, J. E. , Stone, T. , & Ng, J. (2020). Cardiovascular drift and maximal oxygen uptake during running and cycling in the heat. Medicine and Science in Sports and Exercise, 52(9), 1924–1932. 32102057 10.1249/MSS.0000000000002324

[eph13813-bib-0099] Xu, F. , & Montgomery, D. L. (1995). Effect of prolonged exercise at 65 and 80% of ${\dot{V}}_{O_{2}\max}$ on running economy. International Journal of Sports Medicine, 16(05), 309–313. 7558528 10.1055/s-2007-973011

[eph13813-bib-0100] Yoshida, T. , & Watari, H. (1996). Effect of passive and active recovery on PCr kinetics. In The physiology and pathophysiology of exercise tolerance. (pp. 67–74). Springer.

[eph13813-bib-0101] Zanini, M. , Folland, J. P. , & Blagrove, R. C. (2024). Durability of running economy: differences between quantification methods and performance status in male runners. Medicine and Science in Sports and Exercise, 56(11), 2230–2240. 38857519 10.1249/MSS.0000000000003499

